# Development of Deep Learning Models for Predicting In-Hospital Mortality Using an Administrative Claims Database: Retrospective Cohort Study

**DOI:** 10.2196/27936

**Published:** 2022-02-11

**Authors:** Hiroki Matsui, Hayato Yamana, Kiyohide Fushimi, Hideo Yasunaga

**Affiliations:** 1 Department of Clinical Epidemiology and Health Economics School of Public Health The University of Tokyo Tokyo Japan; 2 Department of Health Services Research Graduate School of Medicine The University of Tokyo Tokyo Japan; 3 Department of Health Policy and Informatics Tokyo Medical and Dental University Graduate School Tokyo Japan

**Keywords:** prognostic model, deep learning, real-world data, acute care, claims data, myocardial infarction, heart failure, stroke, pneumonia

## Abstract

**Background:**

Administrative claims databases have been used widely in studies because they have large sample sizes and are easily available. However, studies using administrative databases lack information on disease severity, so a risk adjustment method needs to be developed.

**Objective:**

We aimed to develop and validate deep learning–based prediction models for in-hospital mortality of acute care patients.

**Methods:**

The main model was developed using only administrative claims data (age, sex, diagnoses, and procedures on the day of admission). We also constructed disease-specific models for acute myocardial infarction, heart failure, stroke, and pneumonia using common severity indices for these diseases. Using the Japanese Diagnosis Procedure Combination data from July 2010 to March 2017, we identified 46,665,933 inpatients and divided them into derivation and validation cohorts in a ratio of 95:5. The main model was developed using a 9-layer deep neural network with 4 hidden dense layers that had 1000 nodes and were fully connected to adjacent layers. We evaluated model discrimination ability by an area under the receiver operating characteristic curve (AUC) and calibration ability by calibration plot.

**Results:**

Among the eligible patients, 2,005,035 (4.3%) died. Discrimination and calibration of the models were satisfactory. The AUC of the main model in the validation cohort was 0.954 (95% CI 0.954-0.955). The main model had higher discrimination ability than the disease-specific models.

**Conclusions:**

Our deep learning–based model using diagnoses and procedures produced valid predictions of in-hospital mortality.

## Introduction

Administrative claims databases have been used widely in clinical and epidemiological studies because they have large sample sizes and are easily available. However, administrative data generally lack clinical information [1,2] and do not distinguish between comorbidities at admission and complications after admission [3]. Risk adjustment is not necessarily feasible in studies that use administrative databases because of the lack of data on disease severity, and inadequate risk adjustment can result in confounding by indications.

Various models to predict in-hospital mortality have been developed using comorbidities recorded in administrative data. On the basis of these models, risk scores have been created and used to adjust for disease severity in clinical and epidemiological studies. However, the validity and usability of these models remain controversial [1,4-6]. For example, the Charlson comorbidity index was developed to predict in-hospital mortality, and is commonly used as a risk adjustment measure to capture levels of morbidity in studies that use administrative claims databases. However, this index only uses information on comorbidities that are recorded in the International Statistical Classification of Diseases and Related Health Problems, 10th revision (ICD-10) system [4].

Previous studies showed that additional clinical information improved the performance of mortality prediction models using administrative databases. In a previous study, we developed a procedure-based prediction model using the Japanese Diagnosis Procedure Combination (DPC) database, a nationwide administrative claims database [7]. However, these previous studies used logistic regression models that included only limited numbers of predictors.

Recent advances in machine learning (including deep learning) methods have made it possible to handle large amounts of information and complex models [8,9]. Machine learning methods allow researchers to input a large number of predictors, and variable selection is performed automatically. Conversely, conventional logistic regression requires variable selection based on the existing knowledge of experts.

Many previous studies have used machine learning to create disease-specific mortality prediction models (including models of heart failure [10], stroke [11], and myocardial infarction [12]), as well as all-patient mortality prediction models [12,13]. Most of these models used electronic health records and test results [9-13]. However, to collect such data from a wide range of medical institutions, it is necessary to standardize the electronic medical records. Furthermore, to use such data for clinical and epidemiological studies, experts in each disease area must manually extract information on predictor variables that are specific to the target disease. These factors make it difficult to standardize and use electronic medical records in a nationwide setting.

In this study, we developed and validated a deep learning–based model for predicting all-patient in-hospital mortality using only administrative claims data (including diagnoses and procedure data), which are uniformly formatted and routinely collected in a nationwide setting. To test the performance of the all-patient model, we also constructed disease-specific models for predicting in-hospital mortality of patients with acute myocardial infarction (AMI), heart failure (HF), stroke, or pneumonia, using common severity indices for each disease subgroup. Then, we compared the prediction abilities between the all-patient model and the disease-specific models for each disease subgroup.

## Methods

### Data Source

We conducted a retrospective cohort study. The data from July 2010 to March 2017 were collected from the DPC database. All the patients in the database were included to maximize the generalizability of the results. During the study period, 1569 hospitals contributed to the database. The patients in the database represented about 50% of all the acute-care inpatients in Japan [14].

The following data are included in the DPC database: age, sex, admission date, discharge date, diagnoses, and procedures (drugs, examinations, and surgical and nonsurgical treatments) for each patient. In the DPC database, comorbidities present at admission are clearly distinguished from complications arising after admission. All diagnoses were recorded using the International Statistical Classification of Diseases and Related Health Problems, 10th revision (ICD-10) codes. Procedure records were coded with Japanese conventional codes.

The DPC database also includes several severity indices, namely, the Killip classification for AMI [15,16], New York Heart Association classification for HF [17], Barthel index score for activities of daily living at admission [18], Japan Coma Scale of consciousness level at admission [19]; and age, dehydration, respiration, orientation, blood pressure (A-DROP), the Japan Respiratory Society community-acquired pneumonia severity index [20,21]. The Japan Coma Scale is used widely in Japan to measure impaired consciousness: a score of 0 indicates alert consciousness; single-digit scores (1, 2, 3) indicate being awake without stimuli; double-digit scores (10, 20, 30) indicate patients can be aroused by some stimuli; and triple-digit scores (100, 200, 300) indicate coma. A-DROP is a system for scoring severity of pneumonia that includes age (men ≥70 years, women ≥75 years), dehydration (serum urea nitrogen ≥21 mg/dL), respiratory failure (oxygen saturation by pulse oximetry ≤90% or PaO_2_ ≤60 mm Hg), orientation disturbance (confusion), and low blood pressure (systolic blood pressure ≤90 mm Hg).

Our study was approved by the Ethics Committee of the University of Tokyo School of Medicine (approval number: 3501-(4)).

### Patient Selection

We extracted the data of inpatients who were discharged from hospitals between July 1, 2010, and March 31, 2017. The study population was divided randomly into a derivation cohort (95%) and a validation cohort (5%). For cases with 1-day hospitalization, the time at which we collected the information for prediction and the time at which the outcome occurred could be simultaneous. Because this could lead to an overestimation of the accuracy of the prediction model, we excluded patients who were discharged or died on the day of hospitalization from the validation cohort.

### Variables

The outcome variable was in-hospital death. For predictive variables, we used patients’ demographic information (age, sex, and history of hospitalization in the 180 days before admission), all the ICD-10-based diagnoses at admission, and all the procedures performed on the day of admission. Age was handled as a continuous variable; the other variables were handled as dichotomous variables (0 or 1). We also extracted the Killip classification [15,16], New York Heart Association classification [17], Barthel index score [18], Japan Coma Scale [19], and the A-DROP score [20,21] as common severity indices for specific diseases from the DPC database.

### Development of the Main Model

We developed a deep neural network model as the main model for predicting in-hospital death for all the patients, using 9 layers with 4 hidden dense layers [22,23]. For this, we used the patients’ demographic information, all the ICD-10–based diagnoses at admission, and all the procedures performed on the day of admission. All the layers had 1000 nodes and were fully connected to adjacent layers. We used a softmax layer with 2 nodes as the output layer [24]. Because the numbers of deceased and alive patients were very different, we weighted the deceased cases with the reciprocal of the proportion of deceased cases (ie, 1/0.045=22.3) [23]. We used stochastic gradient descent to obtain neural network weights iteratively [25]. To avoid overfitting, 20% drop-out layers were sandwiched within each of the dense layers and an early stopping procedure involving learning steps using 3% data in the derivation cohort was employed [26]. Details of the weight optimization process are described in Multimedia Appendix 1.

### Development of the Disease-Specific Models

We constructed disease-specific models for predicting in-hospital mortality in subgroups with AMI, HF, stoke, or pneumonia. The 4 models included patient backgrounds (age, sex, and history of hospitalization in the 180 days before admission) and diagnoses, and none of the models included procedures. For the AMI-specific model, we selected patients with AMI and included the Killip classification [15,16]. For the HF-specific model, we selected patients with HF and included the New York Heart Association classification [17]. For the stroke-specific model, we selected patients with stroke and included the Barthel index and the Japan Coma Scale at admission [18,19]. For the pneumonia-specific model, we selected patients with pneumonia and included the A-DROP score [20,21].

### Comparing Prediction Abilities Between the Main Model and the Disease-Specific Models

We applied the main model to the subgroups of patients with AMI, HF, stoke, and pneumonia and compared its prediction performance with the prediction performances of the disease-specific models for AMI, HF, stoke, and pneumonia. We evaluated the performance of each model by calculating performance measures in the validation cohort. Performance measures included the area under the receiver operating characteristic curve (AUC), used to determine the discriminatory ability of the model. We calculated the 95% CI of the AUC using the DeLong method [27] and plotted a calibration curve to determine goodness of fit. We also calculated sensitivity, specificity, and positive and negative predictive values at the threshold determined by the Youden Index method [28]. We obtained CIs for all the indices with 2000 bootstraps.

We also examined whether the risk scores calculated by the disease-specific models improved the discrimination ability of the risk scores calculated by the main model. We incorporated the risk scores calculated by the main and disease-specific models into predictor variables of a logistic regression model and calculated combined risk scores that predicted in-hospital mortality for each disease population. The discrimination ability of the combined risk score was evaluated by its AUC and compared with the AUC of the main model. CIs for the AUC and hypothesis testing for the difference between the main model risk score and combined risk score were calculated using the DeLong method.

## Results

**Figure 1:**
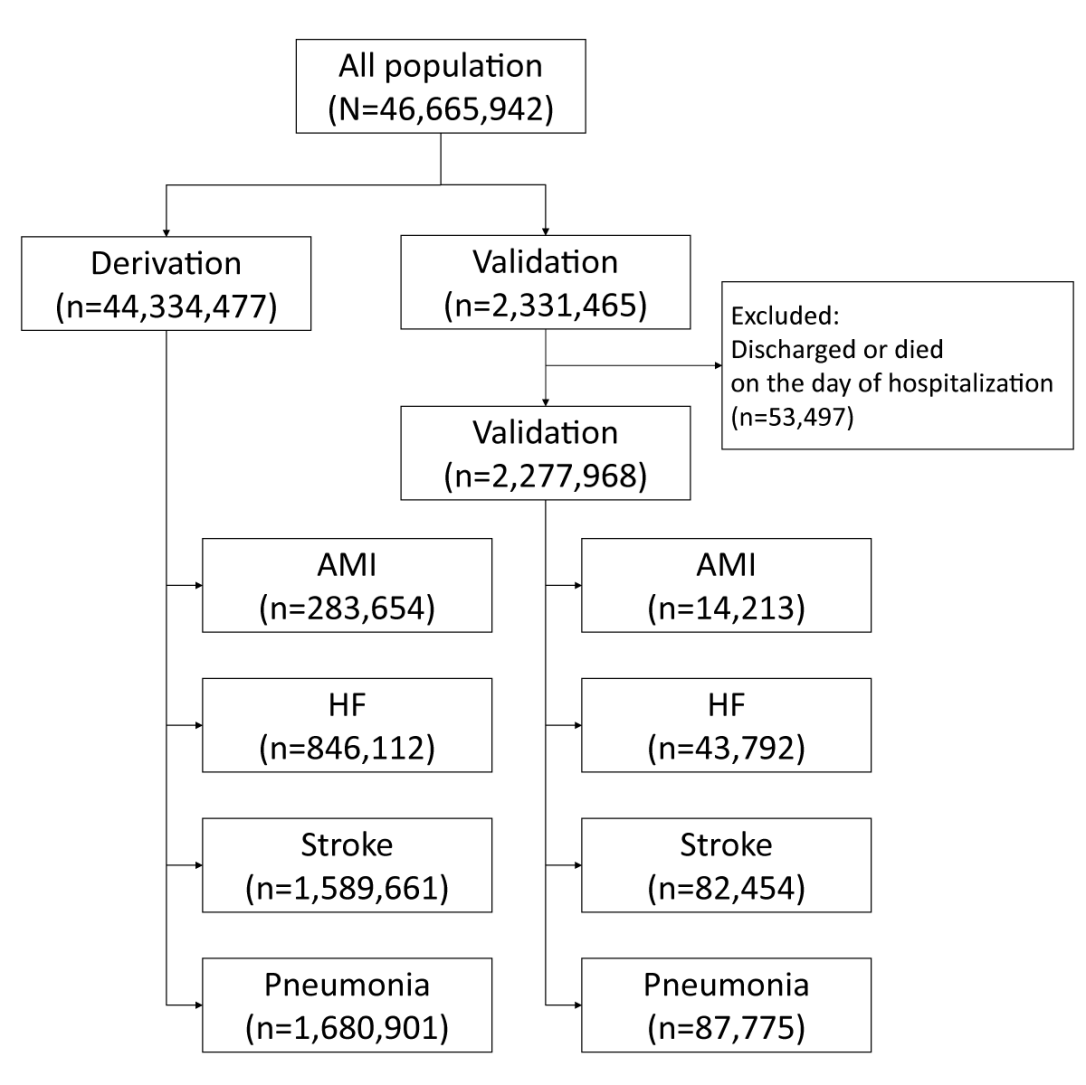
Numbers of patients in the derivation and validation cohorts and disease-specific subgroups. AMI: acute myocardial infarction, HF: heart failure.

We obtained the data for 46,665,942 patients during the study period from the DPC database and divided them into derivation (n=44,334,477) and validation (n=2,331,465) cohorts. We excluded patients from the validation cohort according to the exclusion criteria, which left 2,277,968 patients in the validation cohort (Figure 1).

The characteristics of the derivation and validation cohorts are shown in Table 1. The average lengths of stay were 14.2 days and 14.5 days and in-hospital mortality was 4.3% and 3.7% in the derivation and validation cohorts, respectively. Patients in the validation cohort were slightly older and had more comorbidities than those in the derivation cohort.

The structure of the main model is shown in Table 2. There were 49,297 predictor variables, including 3 variables on patient demographics and history (age, sex, history of hospitalization in the 180 days before admission), 19,930 diagnoses at admission, and 29,364 procedures (drugs, examinations, surgical and nonsurgical treatments). Overall, 52,302,002 weights (=49,297 × 1000 + 1001 × 1000 + 1001 × 1000 + 1001 × 1000 + 1001 × 2) of links between the layers were optimized in the derivation. The script for the deep learning model including model weights is available on our website [29].

**Table 1:** Characteristics of the patients in the derivation and validation cohorts.

Characteristic			Derivation cohort (n=44,334,477)	Validation cohort (n=2,277,968)	*P* value	
Death, n (%)			1,905,286 (4.3)	83,292 (3.7)	<.001	
Length of hospital stay (days), mean (SD)			14.2 (24.1)	14.5 (24.2)	<.001	
Age (years), mean (SD)			60.1 (24.4)	60.4 (24.2)	<.001	
Sex (male), n (%)			23,480,628 (53.0)	1,207,886 (53.0)	.07	
History of hospitalization within 180 days, n (%)			12,282,386 (27.7)	632,362 (27.8)	.07	
**Charlson comorbidity index, n (%)**						<.001
	0-1	28,734,890 (64.8)		1,465,779 (64.3)		
	2-3	11,432,403 (25.8)		594,500 (26.1)		
	≥4	4,165,579 (9.4)		217,605 (9.6)		

**Table 2:** Structure of the main model.

Layer	Input (nodes)	Output (nodes)	Weights, n
1: Input	49,297	1000	49,297,000
2: Drop-out	N/A^a^	N/A	N/A
3: Hidden 1	1001	1000	1,001,000
4: Drop-out	N/A	N/A	N/A
5: Hidden 2	1001	1000	1,001,000
6: Drop-out	N/A	N/A	N/A
7: Hidden 3	1001	1000	1,001,000
8: Drop-out	N/A	N/A	N/A
9: Output	1001	2	2002
Sum of weights	N/A	N/A	52,302,002

^a^N/A: not applicable.

An overview of the main and disease-specific models used in this study is given in Table 3. The total number of weights was calculated as follows: total number of weights = the number of input nodes × 1000 + 1001 × 1000 + 1001 × 1000 + 1001 × 1000 + 1001 × 2.

The AUC of the main model in the validation cohort was 0.954 (95% CI 0.9537-0.9547). The sensitivity, specificity, and positive and negative predictive values at the cutoff point (0.0435) determined by the Youden index method of the main model were 0.920 (95% CI 0.915-0.924), 0.855 (95% CI 0.852-0.860), 0.195 (95% CI 0.192-0.199), and 0.996 (95% CI 0.996-0.997), respectively (Table 4).

**Figure 2:**
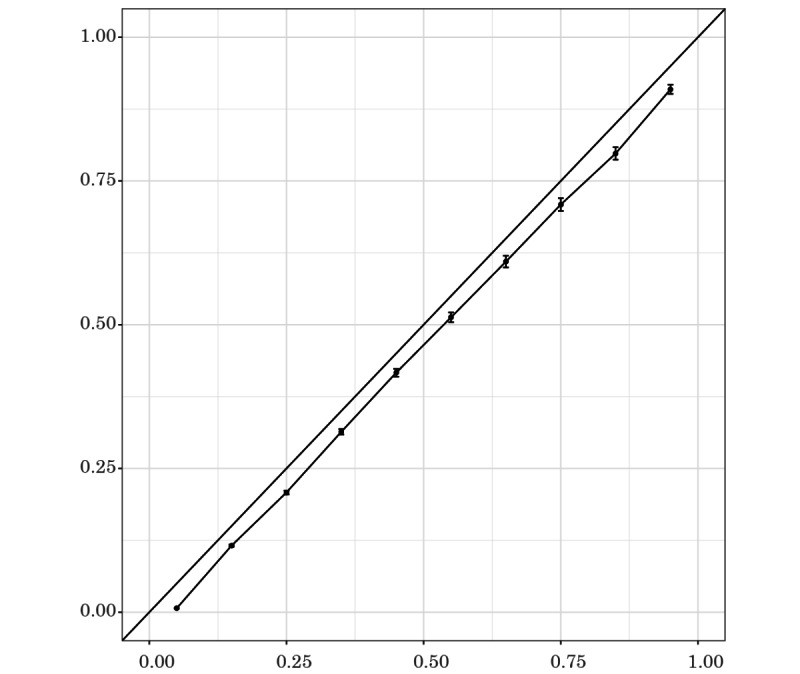
Calibration curves for the observed and estimated mortality in the validation cohort with the main model. X-axis indicates predicted mortality and Y-axis indicates actual mortality.

The calibration curves of the observed and estimated mortality in the validation cohort are shown in Figure 2. Observed and estimated mortality were strongly correlated, but the estimated mortality was slightly lower than the observed mortality.

The AUCs and other prediction metrics of the main and disease-specific models are shown in Table 4. The AUCs of the main model for the AMI, HF, stroke, and pneumonia subgroups were 0.944, 0.832, 0.921, and 0.918, respectively. The AUCs of the disease-specific models for the AMI, HF, stroke, and pneumonia subgroups were 0.876, 0.745, 0.894, and 0.863, respectively. The main model showed significantly higher discriminant ability than the disease-specific models for all 4 subgroups.

**Table 3:** Summary of the main and disease-specific models.

Model	Input (nodes)	Weights, N
Main model	49,297	52,302,002
Acute myocardial infarction model	9	3,014,002
Stroke model	54	3,059,002
Heart failure model	9	3,014,002
Pneumonia model	9	3,014,002

**Table 4:** Performances of the main and disease-specific models.

			AUC^a^ (95% CI)		Threshold		Sensitivity (95% CI)		Specificity (95% CI)		PPV^b^ (95% CI)		NPV^c^ (95% CI)
**Validation cohort (n=2,331,465)**													
	Main model	0.954 (0.954-0.955)		0.0435		0.920 (0.915-0.924)		0.855 (0.852-0.860)		0.195 (0.192-0.199)		0.996 (0.996-0.997)	
**Acute myocardial infarction (n=14,213)**													
	Main model	0.944 (0.938-0.950)		0.087		0.888 (0.864-0.947)		0.862 (0.796-0.881)		0.334 (0.264-0.363)		0.990 (0.988-0.995)	
	Disease-specific model	0.876 (0.866-0.887)		0.087		0.837 (0.797-0.877)		0.783 (0.745-0.817)		0.233 (0.210-0.257)		0.984 (0.981-0.988)	
**Heart failure (n=43,792)**													
	Main model	0.831 (0.825-0.837)		0.118		0.782 (0.729-0.813)		0.719 (0.678-0.771)		0.220 (0.205-0.245)		0.970 (0.965-0.973)	
	Disease-specific model	0.745 (0.738-0.753)		0.097		0.727 (0.678-0.754)		0.642 (0.613-0.688)		0.172 (0.166-0.184)		0.958 (0.954-0.961)	
**Stroke (n=82,454)**													
	Main model	0.921 (0.918-0.925)		0.091		0.863 (0.847-0.901)		0.824 (0.781-0.837)		0.267 (0.234-0.279)		0.988 (0.987-0.991)	
	Disease-specific model	0.894 (0.890-0.898)		0.080		0.824 (0.805-0.836)		0.800 (0.793-0.818)		0.235 (0.229-0.249)		0.984 (0.983-0.985)	
**Pneumonia (n=87,775)**													
	Main model	0.918 (0.915-0.920)		0.075		0.913 (0.896-0.925)		0.769 (0.762-0.786)		0.209 (0.204-0.219)		0.993 (0.991-0.994)	
	Disease-specific model	0.863 (0.859-0.867)		0.064		0.851 (0.809-0.913)		0.705 (0.638-0.744)		0.160 (0.143-0.173)		0.986 (0.983-0.991)	

^a^AUC: area under the receiver operating characteristic curve.

^b^PPV: positive predictive value.

^c^NPV: negative predictive value.

The discriminatory ability of the combined risk scores and the risk scores calculated by the main model are shown in Table 5. All combined risk scores except the one for AMI had significantly higher AUCs than the main model risk scores. However, the differences between the main model risk scores and the combined risk scores were small.

**Figure 3:**
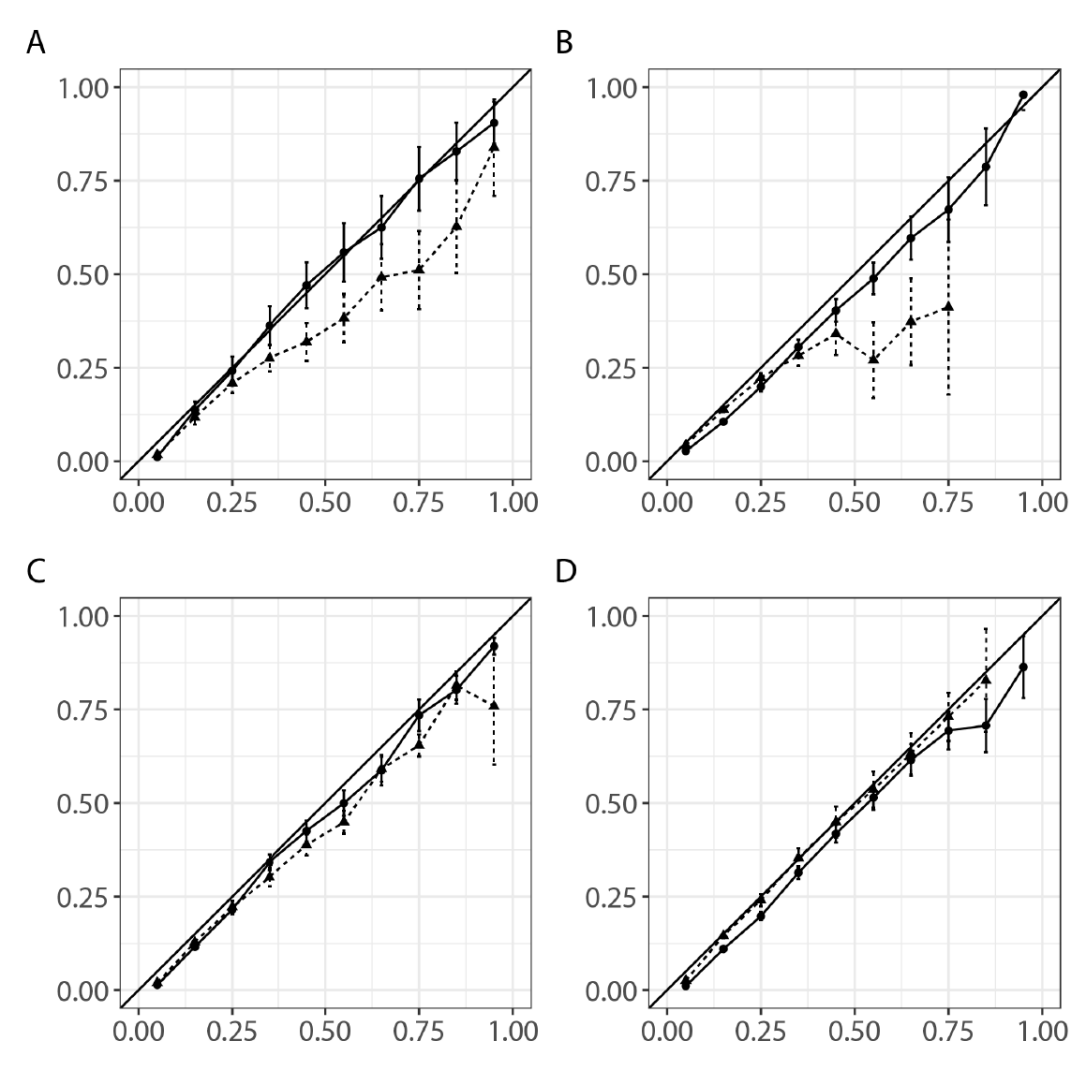
Calibration curves for the observed and estimated mortality in the validation cohort with the disease-specific models. Models for (A) acute myocardial infarction, (B) heart failure, (C) stroke, and (D) pneumonia. X-axis: predicted mortality. Y-axis: actual mortality. Solid line: main model. Dotted line: disease-specific models.

The calibration curves for the main and disease-specific models for the subgroups are shown in Figure 3. The correlations between the observed and estimated mortality were better with the main model than with the disease-specific models for the AMI, HF, and stroke subgroups (Figure 3A-C). For the pneumonia subgroup, the correlations were similar between the main and disease-specific models when the predicted mortality was ≤0.8. However, the disease-specific model failed to estimate mortality well when the predicted mortality was ≥0.8 (Figure 3D).

**Table 5:** Comparison of the discriminatory ability of the combined risk scores and the risk scores calculated by the main model.

	Main model AUC^a^ (95% CI)	Combined risk score AUC (95% CI)	*P* value
Acute myocardial infarction	0.944 (0.938-0.950)	0.945 (0.939-0.951)	.23
Heart failure	0.831 (0.825-0.837)	0.838 (0.832-0.844)	<.001
Stroke	0.921 (0.918-0.925)	0.927 (0.924-0.930)	<.001
Pneumonia	0.918 (0.915-0.920)	0.921 (0.918-0.924)	<.001

^a^AUC: area under the receiver operating characteristic curve.

## Discussion

### Principal Findings

We constructed deep leaning–based prediction models for in-hospital mortality using a large Japanese inpatient database. Patient backgrounds, diagnoses, and treatments on the first day of admission were entered into the models. The overall discriminant abilities of the models were high in subgroups of patients with AMI, HF, stroke, and pneumonia. The main model had better discriminant abilities than disease-specific models using common severity indices. We integrated the risk scores for the main and disease-specific models and calculated combined risk scores. However, the improvement in the predictive performance of the combined risk scores over that of the main model risk scores was only slight.

Risk scores derived from administrative claims databases have been developed previously. For example, the Charlson and Elixhauser models, which use comorbidity information to predict long-term survival, have been used for risk adjustment in clinical and epidemiological studies [30,31]. In this study, a new prediction model for in-hospital mortality developed using administrative claims data showed high discriminatory power (AUC=0.945). We believe that our model can also be used for risk adjustment in clinical and epidemiological studies using administrative claims data that includes diagnoses and procedures.

In a previous study, we constructed a prediction model for in-hospital mortality that incorporated comorbidities and several selected procedures (blood tests, radiography, echocardiogram) on the day of admission [7]. However, that model lacked generalizability; for example, it was not applicable to critically ill patients. The newly constructed model can be used for risk prediction and adjustment for patients with a wide range of disease severity.

In a previous study, the predictive abilities of models with administrative claims data alone were compared with those of models with electronic medical records combined with administrative claims data [32]. The predictive abilities of the models with electronic medical records were higher because the electronic medical records included detailed information related to each patient, such as blood test results, vital signs, and admission data collected during the first 2 days of the index admission.

In this study, a deep learning model that used only massive administrative data had higher predictive ability than models that used disease-specific severity information. On the basis of our results, we consider that large-scale administrative data can be used to predict in-hospital mortality more accurately than the generally used severity indices. Kharrazi et al [33] reported that obtaining information from both administrative data and electronic health records increased the prediction accuracy of their model compared with using each data source alone. Zeltzer et al [32] found that feeding the electronic health record information collected during hospitalization, in addition to the administrative data and pre-hospitalization electronic health record information, into their model resulted in more accurate mortality risk assessment. Rajkomar et al [9] predicted in-hospital mortality with the same level of accuracy as we achieved in this study by using information from structured electronic health records. We also found that a combined risk score, obtained by integrating the main model with a disease-specific model, showed higher prediction accuracy than the risk score obtained from the main model. However, in this study, the difference between the main model and the combined risk score was small, and there was no significant difference between the two risk scores for AMI. This indicates that the main model was able to construct a risk score comparable to the combined risk score even without disease-specific severity information. Therefore, we propose that patient outcome studies can be conducted using administrative data alone, such as the initial hospitalization process and diagnosis, without the need for data on disease severity.

It is not easy to collect electronic health record information in a standardized way and use it for research. We believe that the results of this study can be used in cases where it is not possible to obtain detailed clinical information, such as disease severity and vital signs, that would be included in an electronic health record.

### Limitations

This study has several limitations. First, we did not conduct an external validation. Second, we did not use a variety of machine learning methods (eg, random forest, lasso regression, XGBoost, and their ensembles), so we could not compare the prediction performance of other machine learning methods. Third, because the database used in this study is for acute hospitalization, we could not obtain data on long-term outcomes. Fourth, model accuracy is not always guaranteed for all diseases, so the applicability of the model to other populations needs to be considered.

### Conclusion

In conclusion, we constructed a deep neural network model to predict in-hospital mortality using all the data on diagnoses and procedures performed on the day of admission in a Japanese administrative claims database. Our model using only administrative claims data showed higher prediction ability than our models using the more generally used severity indices. We propose that prognostic models using data on diagnoses and procedures obtained only from administrative claims databases can predict in-hospital mortality and can be used for risk adjustment in clinical and epidemiological studies.

## Appendix

Multimedia Appendix 1 Model weight optimization process details.

## Abbreviations

A-DROP: age, dehydration, respiratory failure, orientation disturbance, and low blood pressure
AMI: acute myocardial infarction
AUC: area under the receiver operating characteristic curve
DPC: Japanese Diagnosis Procedure Combination
HF: heart failure
ICD-10: International Statistical Classification of Diseases and Related Health Problems, 10th revision
NPV: negative predictive value
PPV: positive predictive value
