# Supplementary material for: Development of Deep Learning Models for Predicting In-Hospital Mortality Using an Administrative Claims Database: Retrospective Cohort Study
Source: JMIR Med Inform. 2022 Feb 11;10(2):e27936. doi: 10.2196/27936 (PMC8881780; doi:10.2196/27936)
Supplement: Multimedia Appendix 1 [file medinform_v10i2e27936_app1.pdf]

Supplementary Material

Model structure

The deep learning model was developed with the following structure.

```
unit_size = 1000
model = Sequential()
model.add(Dense(input_dim=Input_unit_nm, units=unit_size))
model.add(Activation('relu'))
model.add(Dropout(0.2))
model.add(Dense(input_dim=unit_size, units=unit_size))
model.add(Activation('relu'))
model.add(Dropout(0.2))
model.add(Dense(input_dim=unit_size, units=unit_size))
model.add(Activation('relu'))
model.add(Dropout(0.2))
model.add(Dense(input_dim=unit_size, units=unit_size))
model.add(Activation('relu'))
model.add(Dropout(0.2))
model.add(Dense(units=2))
model.add(Activation('softmax'))
```

Defining the loss function and setting up an optimization technique

We set the loss function to 'sparse_categorical_crossentropy' and the optimization method to 'sgd'.

```
model.compile(loss = 'sparse_categorical_crossentropy',
            optimizer = 'sgd',
            metrics = ['accuracy'])
```

Model learning process

We defined the learning process of the main model as follows. Because the numbers of dead and alive patients were very different, we weighted the dead numbers with the reciprocal of the proportion of dead cases (that is, 1/0.045=22.3). The processes were divided into 86,000 steps per epoch. Data

from the training data were obtained in a random order of 500 cases per step, and training was conducted by parallel processing. To avoid overlearning, we ran the validation process for each epoch in the following way, and terminated the learning when the progress was no longer observed. After completing one epoch, the learning process was evaluated. When the loss function decreased compared with the previous epoch, the learning was progressed to the next epoch. When the number of epochs reaches 50, the learning was terminated.

```
class_weight = {0:1, 1:22.3}
model.fit_generator(sql_batch_generator(batch_size=500,train_bgn=0,train_
end= 0.92,split_size= 0.10)
                  , steps_per_epoch=86000
                  , epochs= 50
                  , callbacks=[es_cb,cp_cb]
                  , class_weight = class_weight
                  , validation_data=sql_batch_generator(batch_size=500,t
rain_bgn=0.92,train_end= 0.95,split_size= 0.03)
                  , validation_steps=2000)
```
